# Immunogenic cell death in colorectal cancer: a review of mechanisms and clinical utility

**DOI:** 10.1007/s00262-024-03641-5

**Published:** 2024-02-14

**Authors:** M. De Silva, B. C. Y. Tse, C. I. Diakos, S. Clarke, M. P. Molloy

**Affiliations:** 1 Bowel Cancer and Biomarker Research Laboratory, Kolling Institute, Royal North Shore Hospital, St Leonards, NSW Australia; 2 Department of Medical Oncology, Royal North Shore Hospital, St. Leonards, NSW Australia; 3 Sydney Medical School, Faculty of Medicine and Health, The University of Sydney, Sydney, NSW Australia

**Keywords:** Immunogenic cell death, Colorectal cancer, Chemotherapy, Damage-associated molecular patterns, Biomarker

## Abstract

Colorectal cancer (CRC) is a major cause of cancer-related morbidity and mortality worldwide. Despite several clinical advances the survival of patients with advanced colorectal cancer remains limited, demanding newer approaches. The immune system plays a central role in cancer development, propagation, and treatment response. Within the bowel, the colorectal mucosa is a key barrier and site of immune regulation that is generally immunosuppressive. Nonetheless, within this tumour microenvironment, it is evident that anti-neoplastic treatments which cause direct cytotoxic and cytostatic effects may also induce immunogenic cell death (ICD), a form of regulated cell death that leads to an anti-tumour immune response. Therefore, novel ICD inducers and molecular biomarkers of ICD action are urgently needed to advance treatment options for advanced CRC. This article reviews our knowledge of ICD in CRC.

**Supplementary Information:**

The online version contains supplementary material available at 10.1007/s00262-024-03641-5.

## Introduction

The global burden of colorectal cancer (CRC) is significant with some of the highest incidence rates observed in Australasia where it is the second leading cause of cancer-related death [1, 2]. Despite improvements in bowel cancer screening, around 20% of patients present with advanced disease with a historical 5-year overall survival (OS) of 13% [2]. Contemporary real-world series have demonstrated an increase in 5-year OS over the past decade to around 25%, with multivariate analyses attributing this to novel treatments for BRAF-mutated and microsatellite unstable (MSI-H) disease, as well as more patients undergoing surgical resection of liver metastases and receiving third-line treatments [3]. Cytotoxic chemotherapy remains the backbone standard systemic treatment for the majority of advanced CRC patients who typically harbour wild-type RAS/RAF and microsatellite stable tumours. The clinical activity of conventional chemotherapy for CRC has been attributed to direct cytotoxic and/or cytostatic effects. However, there is increasing understanding that many of these treatments induce immunogenic cell death (ICD), a type of regulated cell death resulting in innate and adaptive anti-tumour immune responses that can enhance therapies [4, 5]. Great interest exists in identifying novel ICD inducers which may unlock the clinical activity of immunotherapeutics which are mostly missing for treating CRC patients. Less emphasis has been placed on the potential utility of ICD molecular features as novel biomarkers across cancers. The focus of this review is to describe the role and known mechanisms of ICD and review existing data regarding ICD biomarkers in CRC.

## Immunogenic cell death and anti-cancer therapy

Cell death is an irreversible, stimulus-specific process which may be classified by morphologic, enzymatic, functional, and immunologic characteristics [6, 7]. Regulated cell death (RCD) is an evolutionarily conserved form of cell death driven by specific gene pathways and plays a crucial role in normal embryologic development and post-embryonic homeostasis [6, 7]. The most well-known RCD is apoptosis which involves controlled degradation of cellular components resulting from activation of proteolytic caspases [7]. Other regulated cell death pathways exist including pyroptosis, ferroptosis and necroptosis [6, 7]. Immunogenic cell death (ICD) refers to a type of RCD that occurs in infected or malignant cells as result of endoplasmic reticulum (ER) stress triggered by specific stimuli leading to an antigen-specific immune response and immunological memory within immunocompetent hosts [4, 5].

**Fig. 1:**
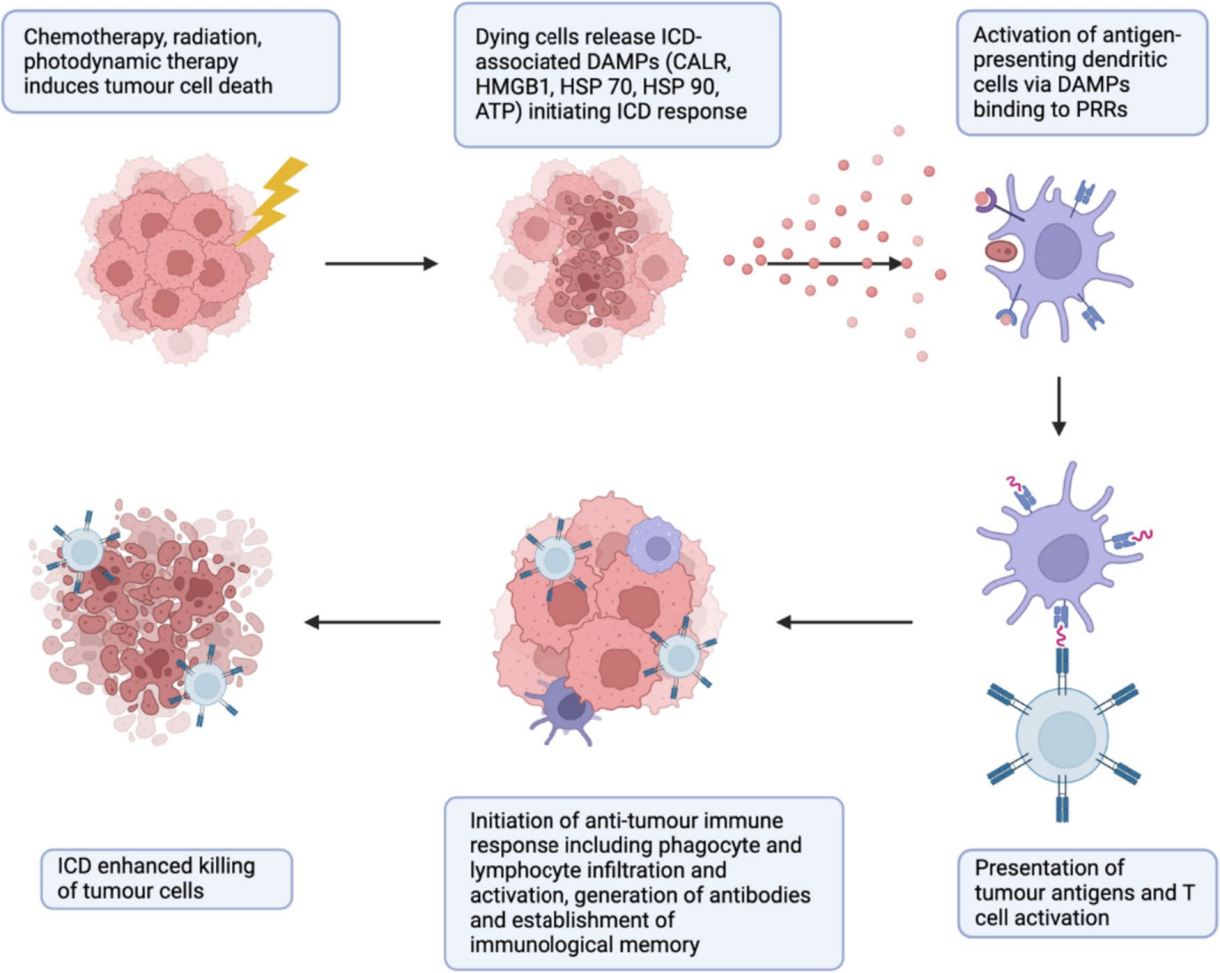
Immunogenic cell death-mediated anti-tumour immune response. Induction of immunogenic cell death (ICD) by chemotherapy, radiotherapy, photodynamic therapy, or oncolytic viruses leading to engagement of adaptive immune response via the spatiotemporally coordinated exposure of immunogenic damage-associated molecular patterns (DAMPs) which bind to their associated pattern recognition receptors (PRRs) on dendritic cells (DCs) or other antigen presenting cells (APCs). This results in increased recruitment, phagocytic activity, and maturation of APCs. Mature APCs then prime cytotoxic T cells (CTLs) within the tumour or regional lymph nodes using tumour-specific antigens (TSA) or tumour-associated antigens (TAAs) leading to a tumour-specific anti-tumour CTL immune response and generation of memory T cells. Figure created with BioRender.com

ICD is characterised by the spatiotemporally coordinated cellular release of immunogenic signals in the form of damage-associated molecular patterns (DAMPs). These are intracellular biomolecules with immunostimulatory features on exposure or secretion by dying cells [8]. Intensive investigation over the past two decades revealed key DAMPs which are hallmarks of ICD [4, 5]. These may be characterised by localisation and stage and include cell surface exposure of the ER resident protein calreticulin (CALR) and heat shock proteins (HSPs), extracellular release of high mobility group box-1 (HMGB1) and pro-inflammatory cytokines, and end-stage degradation factors including ATP, DNA and RNA [4, 5]. DAMPs bind to pattern recognition receptors (PRRs) on antigen presenting cells (APCs); dendritic cells, macrophages and B-cells, leading to activation of both innate and adaptive immune responses [8]. In the malignant context, ICD results in increased APC maturation, migration, and phagocytic activity [4, 5, 8]. Mature APCs then cross-prime cytotoxic T cell lymphocytes (CTLs) within the tumour microenvironment or regional lymph nodes using tumour-specific antigens (TSAs) and tumour-associated antigens (TAAs) leading to a tumour-specific CTL-mediated response and generation of memory T cells (Fig. 1) [4, 5, 8].

It is important to recognise that host immune-tumour interactions through immune-surveillance and dynamic immune-editing play a key role in determining the behaviour of malignant disease and overall patient outcomes [9, 10]. In fact, a major hallmark of malignancy is immune-evasion with malignant cells harnessing multiple strategies to avoid immune-mediated destruction [9]. The tumour microenvironment is typically immune-suppressive and, ultimately, the ability of ICD to initiate and execute adaptive anti-tumour immunity will depend on intrinsic host and tumour factors, not just the initiating ICD agent.

Conventional anti-cancer agents and radiation were thought to control tumour progression solely through direct cytotoxic or cytostatic effects; however, this paradigm was revised following initial observations in multiple murine models demonstrating enhanced efficacy of conventional anti-cancer therapies when human cancer cells were xenografted into immunocompetent mice as opposed to immunodeficient mice [11]. Various physiochemical cellular stressors are associated with ICD including obligate intracellular pathogens, ionizing radiation, photodynamic therapy, and various chemotherapeutics including cytotoxic chemotherapy (i.e. anthracyclines, cyclophosphamide), targeted therapies, and epigenetic modifiers [4, 5]. With increasing appreciation that the activity of many oncologic therapies relates to their ability to induce ICD, there is significant interest in exploiting the concept with novel therapies and/or combinations for deeper and more durable responses.

## Cellular mechanisms of ICD—unfolded protein response, integrated stress response, and ICD

**Fig. 2:**
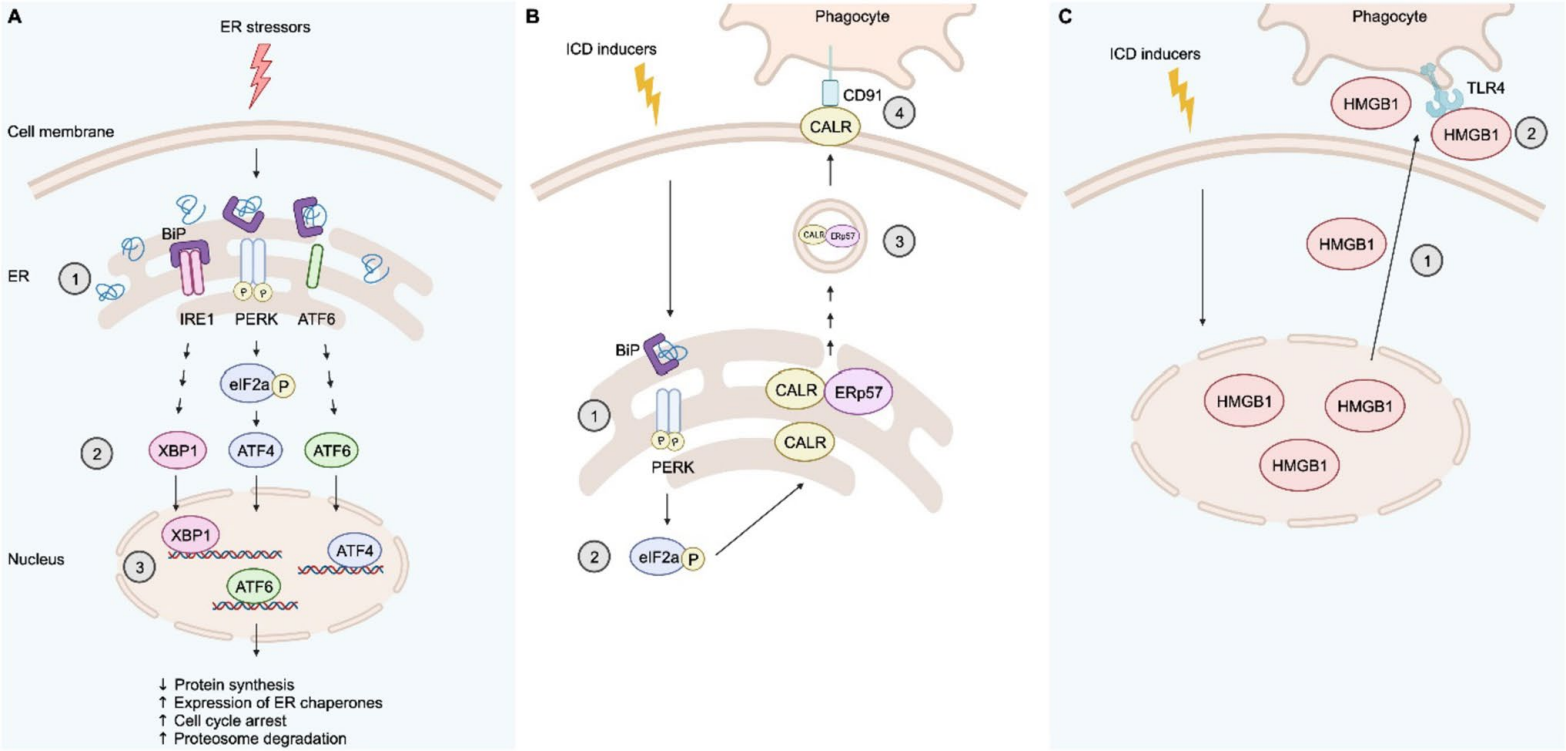
Unfolded protein response and cellular mechanisms of ICD. **A**: Unfolded protein response (UPR); A1: ER stress resulting in accumulation of misfolded proteins which bind BiP leading to dissociation from IRE1, PERK and ATF resulting in transactivation. A2: Downstream activation of XBP1, ATF4 and ATF6 by mRNA splicing, phosphorylation by eIF2α, and proteolytic cleavage within the Golgi apparatus, respectively. A3: Translocation of remodelled XBP1, ATF4 and ATF6 into the nucleus to induce integrated stress response. **B**: CALR signalling in ICD; B1: ER stress by ICD inducer results in activation of PERK. B2: eIF2α phosphorylation triggers CALR co-transport with ERp57 from ER to cell membrane (exact mechanisms unknown). B3: CALR/ERp57 reach cell membrane via SNAP/SNARE vesicular exocytosis. B4: CALR binds with ligand CD91 on APCs triggering the immune response. **C**: HMGB1 signalling in ICD. C1: Dying cells succumbing to ICD passively release HMGB1 from the nucleus into the extracellular space where it binds with ligand TLR4 on APCs triggering the immune response. Figure created with BioRender.com

Study into the underlying mechanisms of ICD revealed the centrality of endoplasmic reticulum (ER) stress. ER stress triggers the unfolded protein response (UPR), a highly conserved pathway and main consequence of cell anoxia (Fig. 2A) [12]. Anoxia interferes with normal protein glycosylation and oxidative protein folding along the protein secretory pathway with subsequent accumulation of misfolded proteins triggering a coordinated cytoprotective response [12]. Three key ER transmembrane receptors are implicated in the relay of signals from ER lumen to the nucleus: Inositol requiring enzyme 1 (IRE1), pancreatic eukaryotic translation initiation factor 2-alpha kinase 3 (PERK/EIF2AK3), and activating transcription factor 6 (ATF6) [12]. Misfolded proteins in the ER lumen bind HSP70 molecular chaperones (BiP/GPR78/HSPA5) which dissociate from IRE1, PERK and ATF6 resulting in transactivation. Ultimately, mature remodelled X-box binding protein 1 (XBP1), activating transcription factor 4 (AFT4) and ATF6 translocate to the nucleus to induce the integrated stress response (ISR) gene expression program [12]. Cytoprotective consequences of UPR and IRS include decreased protein synthesis, increased expression of ER chaperones, cell cycle arrest (G1 phase), and increased proteosome degradation capacity. If homeostasis is not reestablished, pro-apoptotic signalling eliminates the chronically stressed cell [12]. The UPR, ISR and ICD share common features; however, intensive in vitro and in vivo experiments have demonstrated that of the three UPR arms, only the PERK pathway resulting in eIF2a phosphorylation is relevant to ICD [13, 14].

In 2007, Obeid et al. first demonstrated pre-apoptotic surface exposure of CALR as a key feature of ICD using CT26 murine colorectal cells [15]. CALR is a multifunctional protein with primary roles of ER chaperone and Ca2 + buffer for cellular proteostasis [14]. Anthracycline-treated cells showed rapid induction of surface CALR leading to phagocytosis by dendritic cells (DCs) within hours, before manifestation of any apoptotic drivers [15]. CALR surface exposure only occurred on cells succumbing to ICD as opposed to immunologically silent death. Blockade of CALR on the surface of mitoxantrone-treated cells using an antibody or CALR knockdown with specific short interfering RNA (siRNA) suppressed DC-mediated phagocytosis both in vitro and in vivo [15]. Recombinant CALR was able to reverse the defect in immunogenicity caused by CALR-specific siRNA (in vivo and in vitro), and recombinant CALR was not able to promote DC maturation ex vivo, confirming surface exposure of CALR as necessary for DC-mediated phagocytosis [15]. Induction of surface CALR and DC-mediated phagocytosis in both intact and enucleated mitoxantrone-treated cells suggested an indirect, non-genotoxic/non-nuclear mechanism [15]. Ultimately, this was found to be mediated by rapid inactivating phosphorylation of eIF2a which triggers CALR translocation to the cell surface in a molecular complex requiring ERp57 (Fig. 2B) [14, 16]. Cell surface CALR serves as an obligate ‘eat me’ signal and binds to low-density lipoprotein receptor-related protein 1 (LRP1 or CD91), the main ER chaperone sensing pattern recognition receptor (PRR) on antigen presenting cells [14]. Importantly, though recombinant CALR adsorbed to the surface of live cells enhances DC-mediated phagocytosis, this had to be combined with a cell death inducer (e.g. mitoxantrone, etoposide) to evoke local and systemic immune responses in vivo [15]. Therefore, surface CALR is necessary but not sufficient for ICD, with additional signals required.

Seminal research by Apetoh et al. demonstrated a separate immunoadjuvant effect of cytotoxic chemotherapy through post-apoptotic release of HMGB1 in various cancer cells lines treated with irradiation, doxorubicin or oxaliplatin [11]. HMGB1 is a non-histone nuclear protein with pleiotropic roles depending on cellular localisation and secreted HMGB1 is considered a hallmark of ICD [11]. First, dying ovalbumin (OVA)-expressing EG7 mouse thymoma cells were fed to bone marrow derived DCs that were wild-type or lacking specific toll-like receptors (TLRs) and the antigen presenting capacity of DCs was assessed by measuring IL-2 production by MHC class I and II-restricted OVA-specific mouse T cell hybridomas [11]. OVA peptides from irradiated or oxaliplatin-treated EG7 tumour cells, but not live tumour cells, were efficiently presented by all but TLR4^-/-^ DCs. Furthermore, pulsing of mouse-derived DCs with a TLR4 inhibitor or fusion protein inhibited the MHC class I-restricted OVA-specific response [11]. These results confirmed TLR4 as a key damage-sensing PRR required for antigen cross-presentation by dying tumour cells. Of various endogenous ligands that bind and stimulate TLR4, only release of HMGB1 was observed following irradiation of EG7 cells or doxorubicin treatment of CT26 cells. To confirm whether HMGB1 in the supernatant of dying cells would interact with TLR4, RAW264.7 macrophage-like cells expressing TLR4 were incubated with supernatants of doxorubicin-treated CT26 cells (containing > 200 ng/ml free HMGB1) or live CT26 cells (containing < 20 ng/ml free HMGB1) and TLR4 immunoprecipitated. The results confirmed secreted HMGB1 binds to TLR4 on APCs (Fig. 2C) [11]. Anti-tumour vaccination efficacy of doxorubicin or oxaliplatin-treated MCA205 sarcoma cells in mice that were TLR4^-/-^ was reduced compared to wild-type mice. Furthermore, pharmacologic inhibition of TLR4 with a cell permeable inhibitor or depletion of HMGB1 using neutralizing antibodies or HMGB1-specific siRNA reduced the efficacy of anti-tumour vaccination [11]. In the treatment of established tumours, CT26 colon cancers, TS/A breast carcinomas, heterotransplanted GOS osteosarcomas and EL4 thymomas progressed with similar kinetics in immunocompetent wild-type, TLR4^-/-^ and *nu/nu* athymic mice; however, chemotherapy or radiotherapy was less effective in TLR4^-/-^ and *nu/nu* mice [11]. Taken together, these findings confirmed the significant immunoadjuvant role of HMGB1 release from dying tumour cells and its interaction with associated PRR TLR4 on APCs as part of ICD [11].

## Hallmarks of ICD as biomarkers in colorectal cancer

Recent studies have shed light on the potential role of ICD features as novel biomarkers in various cancers [8]. Here, we summarise the literature exploring ICD molecular features as biomarkers in CRC, with a focus on CALR and HMGB1.

### CALR

Touquet et al. investigated CALR expression in 58 naïve human colon adenocarcinomas and paired normal mucosa [17]. Reduced CALR expression was observed compared to normal epithelium at a distance of 10 cm (30/58), and there was an association between CALR expression and mucinous differentiation (*p* = 0.006), but no other pathologic features [17]. In contrast, Vougas et al. using two-dimensional electrophoresis and immunohistochemistry demonstrated increased CALR expression in naïve colorectal tumours compared to adjacent healthy mucosa, plus an association between increased CALR expression and poorly differentiated tumours and disease stage [18]. Neither study correlated CALR expression with recurrence-free or overall survival.

Peng et al. showed an association between increased CALR expression and CD45RO + memory T cell infiltration (*p* = 0.01), and improved 5-year overall survival amongst 68 patients with resected stage IIIB colorectal carcinoma (*p* = 0.022) [19]. The expression of CALR was lower in tumour compared to adjacent normal tissue and there was no association between CALR expression and tumour location or differentiation grade [19].

Ryan et al. similarly explored expression of various ER stress proteins including calnexin, CALR, GRP78 and GRP94 in 23 resected stage II and III colon cancers [20]. Tumour ER stress protein expression was similar to healthy matched samples. Increased calnexin tumour to normal tissue ratio was associated with poor outcomes defined by mortality or recurrence within 4 years of surgery (*p* = 0.0055). However, no such difference was observed in the tumour to normal ratios of CALR, GRP78 or GRP94 [20].

Considering the metastatic setting, Laengle et al. showed that CALR expression was associated with increased recurrence-free survival (RFS) in 33 neoadjuvant-treated colorectal liver metastases (CRLM) patients (HR 0.83, 95% CI 0.69–0.98, *p* = 0.03) [21]. In this retrospective study, the majority of patients had left sided primary tumours (26/33) and synchronous CRLM as opposed to metachronous (19 vs. 14). All patients received at least 3 cycles of chemotherapy (median 5) with a view to curative intent resection. The most common treatment regimen was XELOX (capecitabine plus oxaliplatin, 19/33). Thirty-one patients also received a monoclonal antibody during treatment (bevacizumab (29) or cetuximab (2)) [21]. All patients were BRAF wild-type, but a significant proportion was KRAS mutated (20/33). Median recurrence-free survival and overall survival were 10 and 4 months, respectively. Of note, there was also a correlation between CALR expression, type II interferon-induced proteins, and cytotoxic T lymphocytes within CRLMs [21].

In summary, the available literature supporting CALR as a biomarker in clinical specimens of colorectal malignancy is limited to mostly treatment naïve early stage disease. Correlation of CALR expression with clinicopathologic features has yielded conflicting results with some studies demonstrating reduced expression in tumour compared to adjacent healthy mucosa and vice versa. Few studies explored CALR as a prognostic biomarker, again with conflicting results. Varied associations here are likely due to small sample size and the lack of treatment with ICD inducers. The limited available data and prognostic signals found by Peng et al. and Laengle et al. certainly warrant further exploration of CALR as a prognostic biomarker, particularly in cohorts who have received prior treatment with ICD inducers (see supplementary Table 1).

## HMGB1 tissue expression

Yao et al. examined the prognostic significance of HMGB1 in 192 naïve colorectal primary tumours [22]. Over-expression of HMGB1 was observed in 55.7% cases and significantly correlated with tumour invasion, lymph node involvement, distant metastases and Duke’s stage, and staining in adjacent normal mucosa was weaker [22]. Patients with higher HMGB1 expression had shorter survival and multivariate analysis suggested HMGB1 may be an independent prognostic factor [22].

Suren et al. also explored the prognostic nature of HMGB1 in 72 naïve CRC surgical and biopsy specimens using IHC [23]. HMGB1 expression was positive in a similar proportion (55.6%) and HMGB1 expression corelated with lymph node involvement, metastasis status, stage, tumour grade, perineural invasion, and lymphovascular invasion, but there was no association between expression and survival [23].

A study by Ueda et al. used quantitative real time PCR and IHC to determine HMGB1 expression in 140 naïve primary colorectal tumours and paired normal tissue obtained at the time of surgery. HMGB1 mRNA expression was higher in CRC tissue compared to normal tissue [24]. The investigators dichotomised the cohort into HMGB1-high and HMGB1-low by the median mRNA level for correlation with clinicopathologic and outcome data. Similarly, high-HMGB1 expression was associated with larger (> 3 cm) tumours, lymphatic invasion and lymph node metastases [24]. High-HMGB1 correlated with poorer overall survival and multivariate analyses confirmed HMGB1 mRNA expression was an independent prognostic indicator in CRC (relative risk, 1.59, *p* = 0.04) [24]. IHC revealed strong HMGB1 expression in CRC tissues, but no analyses were performed to correlate intensity or pattern of staining with clinicopathologic features or outcomes [24].

In 2020, Wang et al. reported on the subcellular localisation of HMGB1 in a large series including 369 CRC patients and 68 colorectal adenoma patients undergoing primary surgical treatment [25]. 20 cases of adjacent normal colon mucosa were assessed for comparison. HMGB1 nuclear expression was present in virtually all cases (> 95%), but strong nuclear expression was higher in adenomas and CRCs compared to normal mucosa [25]. The rate of cytoplasmic expression of HMGB1 was higher in CRC specimens compared to adenomas (25.2 vs. 11.8%, *p* < 0.01). Cytoplasmic HMGB1 expression was associated with poor tumour grade, but no other clinicopathologic characteristics in the CRC group [25]. Unlike the prior studies, strong nuclear expression corelated with improved estimated 5-year overall survival compared to low nuclear expression. A similar trend was observed for recurrence-free survival but failed to reach significance (*p* = 0.054). In contrast, strong cytoplasmic expression showed the opposite trend with worse estimated 5-year overall survival and recurrence-free survival [25].

Recently, Porter et al. explored the relationship between HMGB1 expression and clinicopathologic plus outcome data. Tissue cores from 650 naïve CRC specimens and 50 matched normal pairs were examined in tissue microarray [26]. 52 colorectal adenomatous polyps, 69 cancerous polyps and 25 normal mucosal biopsies were also analysed with IHC. Normal colonic epithelium observed strong nuclear staining only, and with progression to cancer, emergence of cytoplasmic staining was observed particularly at the leading edge of cancer polyps with reduced nuclear staining [26]. Strong cytoplasmic staining was associated with lymph node positivity. Of note, HMGB1 expression was associated with features of a cold immune environment with greater FOXP3 + and ICOS + T cell density and reduced cytotoxic CD8 + T cell density, but did not correlate with survival [26].

Two studies explored HMGB1 expression in CRC patients undergoing treatment in the adjuvant and neoadjuvant settings. Peng et al. used IHC and flow cytometry to investigate the relationship between HMGB1 expression, T cell infiltration, and prognosis in 72 patients with resected stage IIIB colorectal carcinoma who received 6 months of adjuvant 5-fluorouracil (5-FU)-based chemotherapy [27]. Most tumours showed only nuclear staining of HMGB1 (53/65) with a smaller cohort exhibiting nuclear and cytoplasmic co-expression (12/65). In normal mucosa, HMGB1 was exclusively nuclear. Stronger HMGB1 expression was associated with increased T cell infiltrate, but nuclear and cytoplasmic co-expression of HMGB1 was inversely associated with T cell infiltrate [27]. HMGB1 expression and localisation were not associated with any other clinicopathologic or outcome data [27].

Using a cohort of 75 early stage low rectal cancers who failed to achieve a complete response to neoadjuvant chemoradiation prior to surgery, Hongo et al. evaluated HMGB1 expression [28]. 52 patients showed high HMGB1 expression, and 23 showed low expression in post-treatment surgical specimens. HMGB1 expression was associated with lymphatic invasion and a lower proportion of well-differentiated tumours. Patients with high HMGB1 expression had significantly poorer response to chemoradiation assessed by tumour reduction ratio and regression grade. However, HMGB1 expression was not associated with recurrence-free or overall survival [28].

Similar to CALR tissue expression, some conflicting results are observed with HMGB1 expression in relatively small retrospective studies with mostly treatment naïve patients. Overall, increased expression is observed in tumour tissue compared to adenomas and healthy mucosa. High HMGB1 expression was associated with unfavourable tumour characteristics and shorter survival. Furthermore, high HMGB1 expression corelated with poorer response to neoadjuvant chemoradiation in rectal cancer patients, albeit in a small cohort who failed to achieve a complete response and there was no assessment of expression pre- and post-treatment (see supplementary Table 2).

## HMGB1 serum expression

Serum HMGB1 has also been explored as a diagnostic and prognostic biomarker. Lee et al. measured serum HMGB1 levels in 219 CRC patients and 75 healthy controls [29]. Average serum HMGB1 levels were 1.5 fold elevated in CRC patients compared to healthy controls, but prognosis was not associated with serum HMGB1 concentration [29]. The researchers did not correlate tumour HMGB1 expression with serum or explore relationship between tumour expression and clinicopathologic or outcome data.

In a prospective observational study, serum HMGB1 was measured 24 and 48 h after radioembolization of CRLM [30]. 49 consecutive CRC patients were assessed for serum HMGB1, RAGE and activity of deoxyribonuclease. Median serum HMGB1 at 24 h was higher in non-responders. Furthermore, high pre-treatment and 24 h HMGB1 levels were associated with poorer survival [30].

Sun et al. conducted a randomised study in patients with CRLM undergoing transarterial chemoembolization (TACE) to explore the predictive ability of serum HMGB1 on post-TACE liver damage and treatment efficacy [31]. Patients were randomly assigned to conventional TACE or drug eluting bead TACE. A significant rise in serum HMGB1 was noted in all patients post-TACE peaking roughly 24 h after treatment. Patients whose serum HMGB1 rose by more than 50% post-TACE had worse progression-free survival [31].

Bains et al. analysed serum HMGB1 in 50 patients with locally advanced rectal cancer undergoing an intensified neoadjuvant protocol with 4 weeks of chemotherapy followed by 5 weeks of chemoradiotherapy, both modalities containing oxaliplatin, followed by resection [32]. Serum HMGB1 was measured pre-treatment, post-chemotherapy and post-chemoradiotherapy. Most patients had T3 or T4 disease (90%) and lymph node involvement (82%). Baseline serum HMGB1 was not associated with any clinicopathologic characteristics. Serum HMGB1 observed a slight increase over the course of multimodal treatment in the whole group [32]. Interestingly, serum HMGB1 rise following induction chemotherapy was a strong predictor of distant metastasis-free survival (DMFS) and overall survival, and the higher the rise in post-chemotherapy HMGB1 from baseline, the lower the risk of metastatic failure [32]. In contrast, patients who developed DMFS events had a non-significant drop in HMGB1 expression following neoadjuvant chemotherapy and a drop in serum HMGB1 at any stage of multimodality treatment was associated with worse outcomes [32].

In summary, the utility of HMGB1 as a biomarker in colorectal cancer has been more thoroughly explored compared to CALR, both in tissue and serum. Serum HMGB1 expression is elevated in CRC patients compared to healthy volunteers. Two studies revealed increased serum HMGB1 post-locoregional treatment of CRLM corelated with worse survival. However, elevated serum HMGB1 may be a reflection of treatment-related liver injury over ICD induction as HMGB1 is known to play an important role in liver ischemic reperfusion injury [31]. The study by Bains et al. is interesting and suggests change in serum HMGB1 over the course of multimodal neoadjuvant treatment for rectal cancer (with ICD inducers – oxaliplatin, radiation) predicts metastatic failure, and this must be explored in a larger cohort of patients (see supplementary Table 2).

## ICD inducers in colorectal cancer

Cytotoxic chemotherapy remains the standard treatment for most colorectal cancer patients in adjuvant and metastatic treatment settings. 5-FU, a pyrimidine analogue that inhibits thymidylate synthase, forms the backbone of most regimens. Oxaliplatin, a covalent nuclear DNA binder, or irinotecan, a topoisomerase I inhibitor, are often combined with 5-FU as FOLFOX (fluorouracil, leucovorin, oxaliplatin), FOLFIRI (fluorouracil, leucovorin, irinotecan) or FOLFOXIRI (fluorouracil, leucovorin, oxaliplatin, irinotecan) for improved efficacy [33, 34]. The ability of oxaliplatin to act as a potent ICD inducer was initially demonstrated by Tesniere et al. using CT26 murine colorectal cells [35]. Both 5-FU and irinotecan also demonstrate ability to induce ICD through cell surface CALR exposure and increased MHC-I expression in mouse and human cancer cell lines [36, 37]. Targeted biologic therapies with an anti-angiogenic (e.g. bevacizumab) or anti-epidermal growth factor receptor (anti-EGFR) monoclonal antibodies (e.g. cetuximab, panitumumab) are also typically added to the chemotherapy backbone for improved efficacy [33]. The ability of bevacizumab to induce ICD has not been well studied. In contrast, cetuximab has been shown to induce ICD either alone or in combination with FOLFIRI in a murine model [38].

Considering various treatment protocols, objective response rates vary between 50 and 65% with median progression-free survival between 8 and 12 months for first-line metastatic treatment, and longer-term survival is poor with systemic treatment alone [34]. Few biomarker-positive patients benefit from newer molecular-targeted treatments such as encorafenib/cetuximab (BRAF-mutant, second or third-line) or checkpoint inhibitors (MSI-H, first-line) [33]. The combination of checkpoint inhibitors and standard cytotoxic ICD inducers (5-FU, oxaliplatin, irinotecan) in microsatellite stable (MSS) colorectal cancer is currently being investigated in trials as this strategy has worked well in other malignancies [39]. The basis for this being increased tumour neoantigen exposure via direct cytotoxic effects of chemotherapy (enhanced tumour antigenicity) with enhanced immune adjuvanticity utilising appropriate ICD inducers (exposure of relevant DAMPs) may unlock the activity of immune checkpoint inhibitors which is currently lacking for most CRC patients. Randomised studies are few, with response rates and progression-free survival similar to or slightly improved compared to standard treatment alone [40, 41]. There is a clear need for novel therapeutics in colorectal cancer and high-throughput screening and validation platforms with a focus on harnessing ICD are essential. Recently, multiple agents have been identified as potential ER stress targeted therapies including ceapins (pyrazole amide ATF6 activators), HHQ-4 and plumbagin (GPR78 inhibitors), GSK2606414 and GSK2656157 (ATP-competitive PERK kinase inhibitors), and bullatacin with promising pre-clinical data emerging [42–47].

## Conclusion

Despite a great deal of interest in ICD, there is limited data monitoring ICD biomarkers in colorectal cancer trials. Greater emphasis is required to explore this in prospective studies with patients who receive ICD inducers and compare pre- and post-treatment expression of ICD biomarkers. More robust ICD biomarker platforms will enable effective identification of novel ICD inducers and lead to more effective use of conventional oncologic therapies, either alone or in combination with newer immunotherapeutic treatments. A yet to be realised benefit from ICD would be the option of less intervention with fewer cycles of cytotoxic chemotherapy, reducing morbidity and associated health costs.

### Supplementary Information

Below is the link to the electronic supplementary material.

Supplementary file1 (DOCX 17 KB)

Supplementary file2 (DOCX 15 KB)

## Data Availability

No datasets were generated or analysed during the current study.
