# Supplementary material for: Immunogenic cell death in colorectal cancer: a review of mechanisms and clinical utility
Source: Cancer Immunol Immunother. 2024 Feb 14;73(3):53. doi: 10.1007/s00262-024-03641-5 (PMC10866783; doi:10.1007/s00262-024-03641-5)
Supplement: Supplementary file 1 — Supplementary file1 (DOCX 17 KB) [file 262_2024_3641_MOESM1_ESM.docx]

| **Ref. no.** | **Authors**  **(date)** | **Study design** | **Number of participants** | **Stage IV patients included - Y/N (n)** | **Biospecimen** | **Compared with paired normal mucosa – Y/N** | **Treatment naïve biospecimen– Y/N** | **Expression associated with any clinico-pathological features – Y/N** | **Expression associated with survival – Y/N** |
| --- | --- | --- | --- | --- | --- | --- | --- | --- | --- |
| 17 | Touquet et al (2007) | Prospective | 58 | Y (9) | Colorectal primary | Y | Y | Y – mucinous differentiation | Not assessed |
| 18 | Vougas et al (2008) | Prospective | 21 | Not stated | Colorectal primary | Y | Y | Y – poor differentiation and disease stage | Not assessed |
| 19 | Peng et al (2010) | Retrospective | 68 | N | Colorectal primary | Y | Y | Y – CD45RO+ memory T cell infiltration | Y – improved 5-year overall survival |
| 20 | Ryan et al (2016) | Prospective | 23 | N | Colorectal primary | Y | Y | N | Not assessed |
| 21 | Leangle et al (2018) | Retrospective | 33 | Y (33) | Colorectal liver metastases | Not applicable | N (neoadjuvant systemic therapy) | Y – type II interferon proteins and CD8 T lymphocytes in CRLMs | Y – increased recurrence-free survival |

**Supplementary table 1:** Summary of studies exploring CALR expression as a biomarker in colorectal cancer.
