# Supplementary material for: Immunogenic cell death in colorectal cancer: a review of mechanisms and clinical utility
Source: Cancer Immunol Immunother. 2024 Feb 14;73(3):53. doi: 10.1007/s00262-024-03641-5 (PMC10866783; doi:10.1007/s00262-024-03641-5)
Supplement: Supplementary file 2 — Supplementary file2 (DOCX 15 KB) [file 262_2024_3641_MOESM2_ESM.docx]

| **Ref. no.** | **Authors**  **(date)** | **Study design** | **Number of participants** | **Stage IV patients included - Y/N (n)** | **Biospecimen** | **Compared with paired normal control – Y/N** | **Treatment naïve biospecimen – Y/N** | **Expression associated with any clinico-pathological features** | **Expression associated with survival – Y/N** |
| --- | --- | --- | --- | --- | --- | --- | --- | --- | --- |
| 22 | Yao et al (2010) | Retrospective | 192 | Y (33) | Colorectal primary | Y | Y | Y – tumour invasion, lymph node involvement, Duke’s stage, and distant metastases | Y – reduced survival |
| 23 | Suren et al (2014) | Retrospective | 72 | Y (16) | Colorectal primary | N | Y | Y – lymph node involvement, metastasis status, stage, tumour grade, perineural and lymphovascular invasion | N |
| 24 | Ueda et al (2014) | Retrospective | 140 | Y (12) | Colorectal primary | Y | Y | Y – larger tumours, lymphatic invasion, and lymph node involvement | Y – reduced overall survival |
| 25 | Wang et al (2023) | Retrospective | 369 | Y (29) | Colorectal primary | Y | Y | Y – poor tumour grade | Y – increased 5-year overall survival with strong nuclear expression. Reduced 5-year overall survival and recurrence-free survival with strong cytoplasmic expression |
| 26 | Porter et al (2023) | Retrospective | 650 | N | Colorectal primary | Y | Y | Y – lymph node involvement and cold immune environment | N |
| 27 | Peng et al (2010) | Retrospective | 72 | N | Colorectal primary | Y | Y | Y – T cell infiltration with nuclear expression | N |
| 28 | Hongo et al (2015) | Retrospective | 75 | N | Colorectal primary | N | N (neoadjuvant chemoradiation) | Y – lymphatic invasion, lower proportion of well differentiated tumours, and poorer response to neoadjuvant treatment | N |
| 29 | Lee et al (2012) | Retrospective | 219 | Y (70) | Serum | Y (serum healthy volunteers) | Y | Y – tumour stage | N |
| 30 | Fahmuller et al (2013) | Prospective | 49 | Y (49) | Serum | N | N (radioembolization of CRLM) | Not assessed | Y – poor survival in patients with high 24-hour pre-treatment serum HMGB. Increased in non-responders |
| 31 | Sun et al (2020) | Prospective randomised | 106 | Y (106) | Serum | N | N (c-TACE vs DEB-TACE for CRLM) | Y – higher tumour differentiation in the DEB-TACE cohort | Y – shorter progression-free survival in patients with >50% rise in serum HMGB post-treatment |
| 32 | Bains et al (2020) | Prospective single arm | 50 | N | Serum | N | N (neoadjuvant chemotherapy and chemoradiotherapy for locally advanced rectal cancer) | N | Y – increased metastases-free and overall survival with rise in serum HMGB1 post induction chemotherapy. Worse outcomes with drop in serum HMGB1 at any stage over multimodal treatment |

**Supplementary table 2:** Summary of studies exploring HMGB1 as a biomarker in colorectal cancer.
